# When Symptomatic Hypercalcaemia and Lymphadenopathy Mimic Malignancy: An Atypical Case of Elderly-Onset Sarcoidosis

**DOI:** 10.7759/cureus.116013

**Published:** 2026-09-09

**Authors:** Raneem E AlKhawaja, Ali M AlAhmed, Ali H AlAali, Aalaa Mubarak

**Affiliations:** 1 Internal Medicine, Salmaniya Medical Complex, Manama, BHR; 2 Pathology, Department of Laboratory and Pathology, Salmaniya Medical Complex, Manama, BHR

**Keywords:** atypical presentation, constitutional symptoms, elderly-onset sarcoidosis, eos, granulomatous disease, hypercalcemia, late-onset sarcoidosis, lymphadenopathy, malignancy mimic, non-caseating granulomas

## Abstract

Sarcoidosis is a multisystem granulomatous disorder most commonly affecting the lung parenchyma and lymph nodes. However, virtually any organ system may be involved, resulting in a broad spectrum of clinical manifestations. Despite being predominantly a disease of younger adults, sarcoidosis has been infrequently reported in individuals older than 65 years, resulting in a limited body of literature on elderly-onset disease. We describe the case of an 83-year-old woman who presented with severe symptomatic hypercalcaemia accompanied by fatigue and unintentional weight loss, raising suspicion for an underlying malignancy. Initial laboratory investigations revealed an elevated serum calcium level of 3.72 mmol/L and a suppressed parathyroid hormone (PTH) level of 0.76 pmol/L, consistent with PTH-independent hypercalcaemia, which prompted an extensive diagnostic evaluation for malignancy. Ultimately, lymph node biopsy demonstrated non-caseating granulomas, consistent with a diagnosis of sarcoidosis. This case underscores the importance of recognising elderly-onset sarcoidosis as a potential mimic of malignancy and maintaining a high index of suspicion to avoid diagnostic delay.

## Introduction

Sarcoidosis is a systemic inflammatory disease of unknown aetiology, characterised by the formation of non-caseating granulomas, primarily involving the lung and lymph nodes [1]. Beyond pulmonary involvement, sarcoidosis may affect multiple extrapulmonary organ systems, including the ocular, cardiac, neurological, cutaneous, hepatosplenic, renal, and musculoskeletal systems [1]. Sarcoidosis is typically observed in young adults, with the majority of cases presenting between 25 and 45 years of age [1]. A female predominance is observed, with a male-to-female ratio of 1:2 [1].

The prevalence of sarcoidosis varies geographically, with the highest reported rates in North European countries such as Sweden, where the estimated prevalence is 140-160 per 100,000 [2]. In contrast, Asian countries such as Japan, South Korea, and Taiwan report a lower prevalence of 1-5 per 100,000 [2]. The prevalence of sarcoidosis in the Kingdom of Bahrain remains unknown, as no published epidemiological studies have reported national prevalence estimates. A 2019 review of sarcoidosis in the Middle East cited data from neighbouring countries, including Saudi Arabia, where a prevalence of approximately 13 per 100,000 was reported in the eastern region [3].

Elderly-onset sarcoidosis, which is defined as disease onset after the age of 65 years, is quite uncommon and associated with worse prognosis and higher mortality [4,5]. A limited number of cases have been documented in the literature of elderly-onset sarcoidosis, accounting for only 7%-15.3% of total cases [4,5]. This subgroup presents unique diagnostic challenges, as its clinical manifestations may overlap with those of malignancy and other age-related conditions.

We report a case of elderly-onset sarcoidosis in an 83-year-old female patient who presented with symptomatic hypercalcaemia, generalised lymphadenopathy, and non-specific constitutional symptoms. A diagnosis of sarcoidosis was established following comprehensive clinical, radiological, and histopathological evaluation.

## Case presentation

An 83-year-old Bahraini woman with a past medical history of hypothyroidism, chronic constipation, and recurrent urolithiasis was brought to the emergency department by her daughter with a one-month history of constipation, right-sided abdominal pain radiating to the back, decreased appetite, and fatigue. She described the abdominal pain as constant and colicky in nature, aggravated by food intake and exacerbated by movement. She also reported a bitter taste in her mouth and an unintentional weight loss of 10 kg over the preceding three months. She had a history of chronic constipation, for which she used laxatives as needed. Her last bowel movement was one day before presentation following the use of lactulose and was soft in consistency. She denied melena, hematochezia, or mucus in the stool. Collateral history obtained from her daughter revealed that she had recently been experiencing symptoms of low mood.

She denied any history of fever, night sweats, headache, cough, shortness of breath, chest pain, dizziness, vomiting, and urinary symptoms, including dysuria or hematuria. She denied any recent travel.

Additionally, her daughter reported that she has an old right-sided rib fracture that caused her chronic pain. Otherwise, her surgical history was unremarkable. Her only long-term medications are levothyroxine 75 mcg daily for her hypothyroidism, and lactulose 30 mL as needed for constipation. Her family history was negative for malignancy or autoimmune diseases. She was a lifelong non-smoker and had never consumed alcohol. Regarding her functional status, she had limited mobility and required assistance with ambulation and activities of daily living. She lived with her daughter, who was her primary caregiver.

In the emergency department, the patient was hemodynamically stable and in no acute distress, with a heart rate of 75 beats/min, blood pressure of 105/80 mmHg, oxygen saturation of 96% on room air, and a temperature of 37.0°C. On physical examination, her abdomen was soft and slightly distended, with mild tenderness in the right upper quadrant and right flank, without guarding or rebound tenderness. Hepatosplenomegaly was noted on palpation. Examination of the remaining systems was unremarkable.

Initial laboratory investigations (Table 1) showed a normal complete blood count and urinalysis. However, her renal function test demonstrated elevated urea and creatinine, at 10.5 mmol/L and 127 μmol/L, respectively. The metabolic panel revealed marked hypercalcaemia, with a serum calcium level of 3.72 mmol/L. A comprehensive evaluation of the underlying cause of hypercalcaemia demonstrated normal serum phosphorus and vitamin D levels, with a suppressed parathyroid hormone (PTH) level, consistent with PTH-independent hypercalcaemia. Ideally, serum calcitriol (active vitamin D) levels would also have been measured; however, the assay was not available at the time. Liver function tests revealed elevated total protein and globulin levels, with a normal albumin level.

**Table 1:** Initial laboratory investigations on admission

Parameter	Reference range (units)	Result
Hematology		
White blood cell count	3.4-9.6 (×10⁹/L)	7.8
Hemoglobin	11.6-15.0 (g/dL)	11.7
Platelet count	150-400 (×10⁹/L)	366
Renal function		
Urea	3.2-8.2 (mmol/L)	10.5
Creatinine	45-90 (μmol/L)	127
Electrolytes	-	Within normal range
Liver function test		
Total protein	57-82 (g/L)	89
Albumin	32-46 (g/L)	39
Globulin	15-30 (g/L)	50
Alkaline phosphatase	56-116 (U/L)	97
Gamma-glutamyl transferase	≤38 (U/L)	33
Alanine aminotransferase	10-49 (U/L)	11
Lactate dehydrogenase	120-246 (U/L)	224
Calcium and bone-mineral profile		
Calcium	2.20-2.55 (mmol/L)	3.72
Phosphorus	0.75-1.65 (mmol/L)	1.20
25-hydroxyvitamin D	≥50 (nmol/L)	84
Parathyroid hormone	1.96-9.33 (pmol/L)	0.76
Endocrine		
Thyroid-stimulating hormone	0.55-4.78 (μIU/mL)	3.88
Inflammatory markers		
Erythrocyte sedimentation rate	≤20 (mm/hour)	120
C-reactive protein	≤9 (mg/L)	4

In terms of initial imaging, the patient underwent erect abdominal and chest radiography in the Emergency Department. The chest radiograph revealed a widened mediastinum (Figure 1). The abdominal radiograph demonstrated significant faecal loading within the right-sided colon (Figure 2).

**Figure 1:**
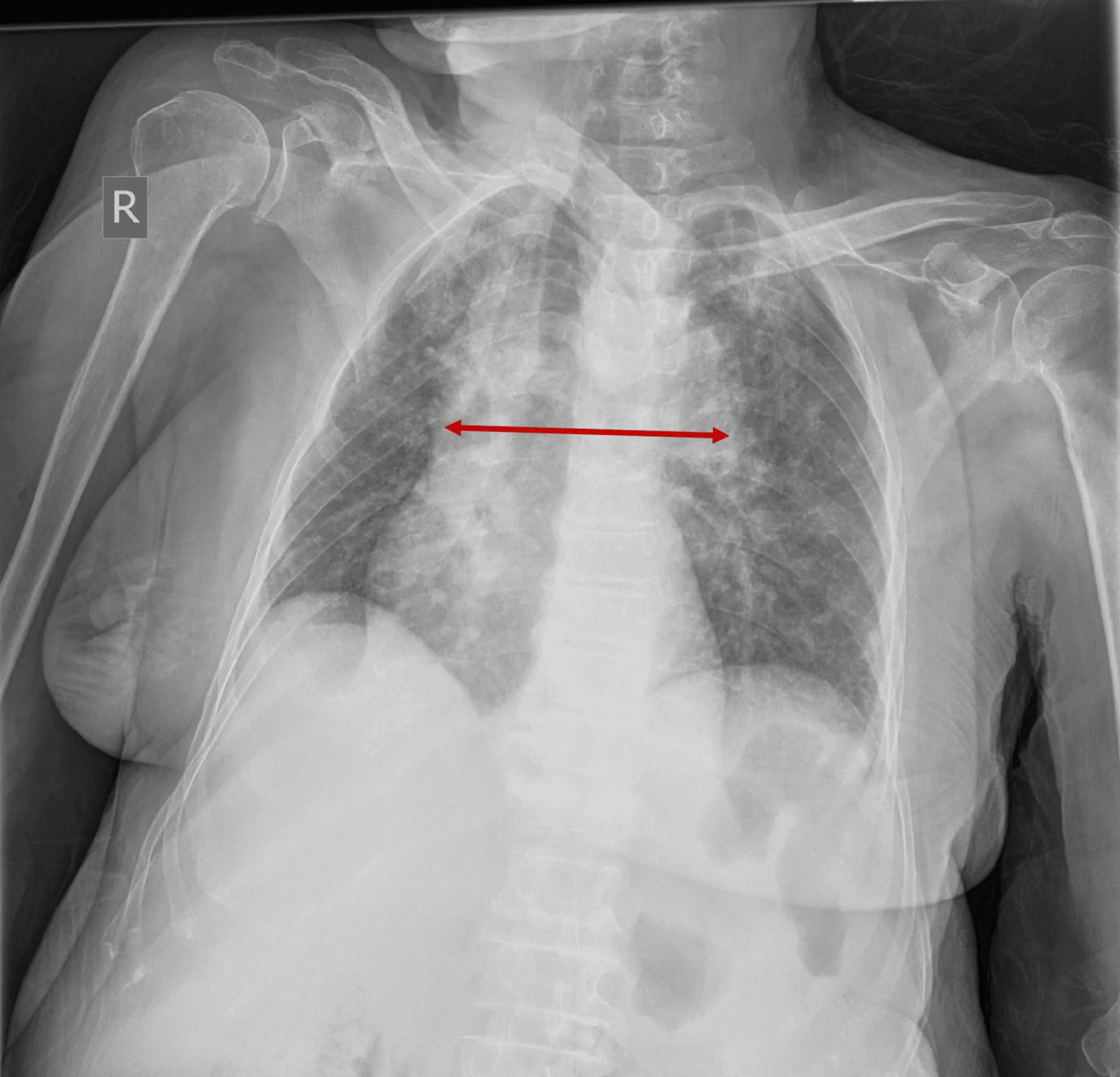
Chest radiograph showing widened mediastinum (red arrow).

**Figure 2:**
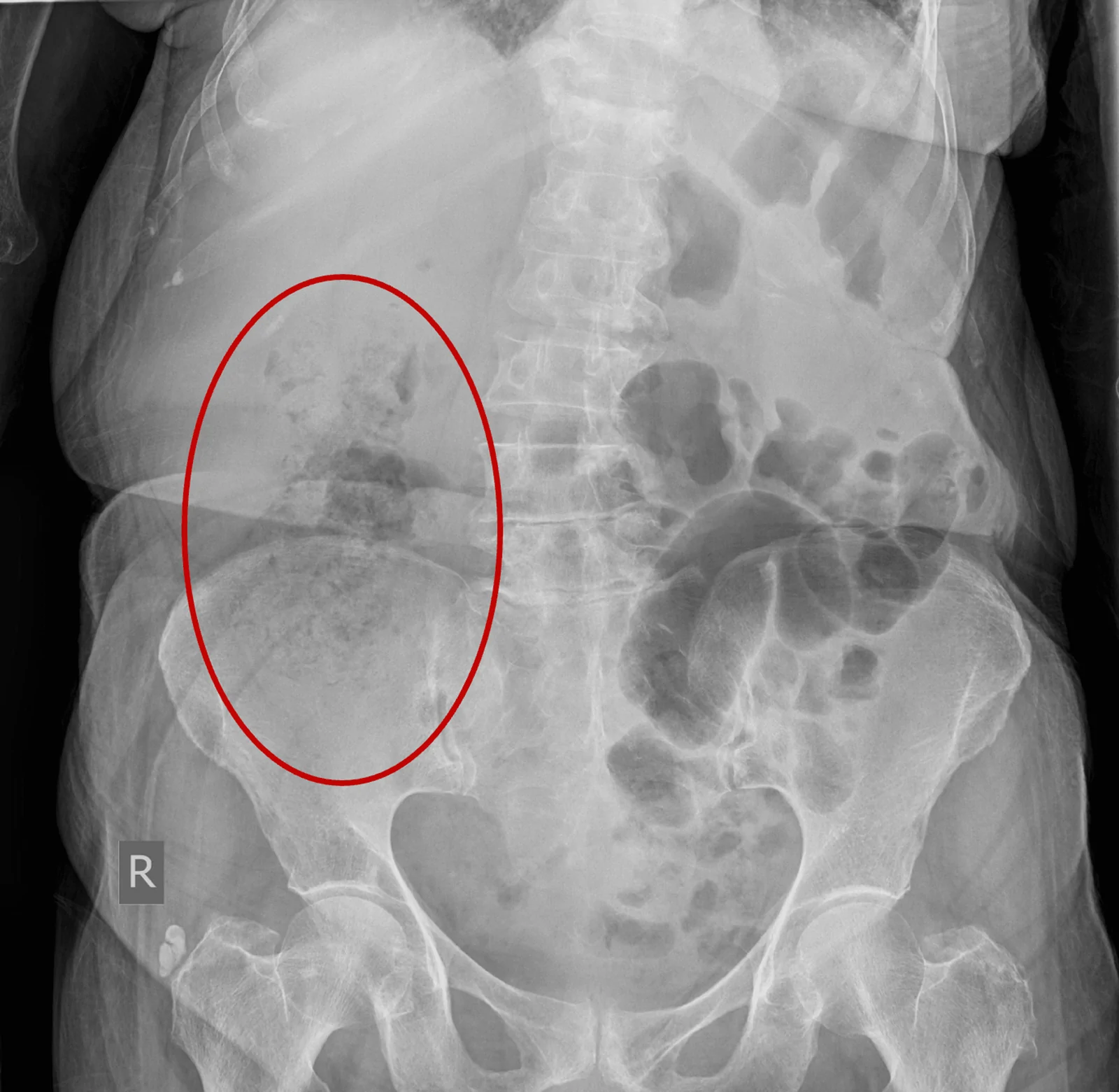
Erect abdominal radiography showing faecal loading predominantly within the right colon (red circle). Dilated bowel loops are observed in the left colon; no radiographic evidence of bowel obstruction or pneumoperitoneum.

The patient was admitted under the General Medicine team with a working diagnosis of symptomatic hypercalcaemia for further evaluation. Acute management was initiated, focusing on symptomatic treatment. For constipation, she was initially administered a sodium phosphate enema, followed by oral lactulose 30 mL and glycerin suppositories as needed. Management of hypercalcaemia included intravenous fluid resuscitation. Given her advanced age, cardiology consultation was obtained to assess her cardiac function and guide the safety of intravenous fluid administration. A bedside transthoracic echocardiogram demonstrated preserved left ventricular systolic function with an ejection fraction of >60%, mild mitral and tricuspid regurgitation, normal right-sided chamber dimensions, a normal-calibre inferior vena cava, and no pericardial effusion. Following cardiology assessment, she received 4 L of intravenous 0.9% sodium chloride over 24 hours, with strict monitoring of fluid input and output. Intravenous furosemide 40 mg daily was administered to mitigate the risk of fluid overload.

Within a few days of admission, her calcium levels reduced to 2.84 mmol/L but still remained above the normal reference range. To address the persistent hypercalcaemia, she was prescribed a single dose of intravenous zoledronic acid 4 mg, after which her calcium level normalised to 2.50 mmol/L within two days. 

During her hospital course, she underwent computed tomography (CT) chest, abdomen, and pelvis scans. The CT abdomen and pelvis showed mild hepatomegaly, with the liver measuring 18 cm, and mild splenomegaly, with the spleen measuring 14 cm. Prominent para-aortic, pelvic, and inguinal lymph nodes were also noted. Furthermore, the chest CT reported multiple enlarged mediastinal lymph nodes, along with bilateral patchy ground-glass opacities, predominantly involving the right upper lobe, with associated mass-like consolidation and fibrotic changes (Figures 3, 4).

**Figure 3:**
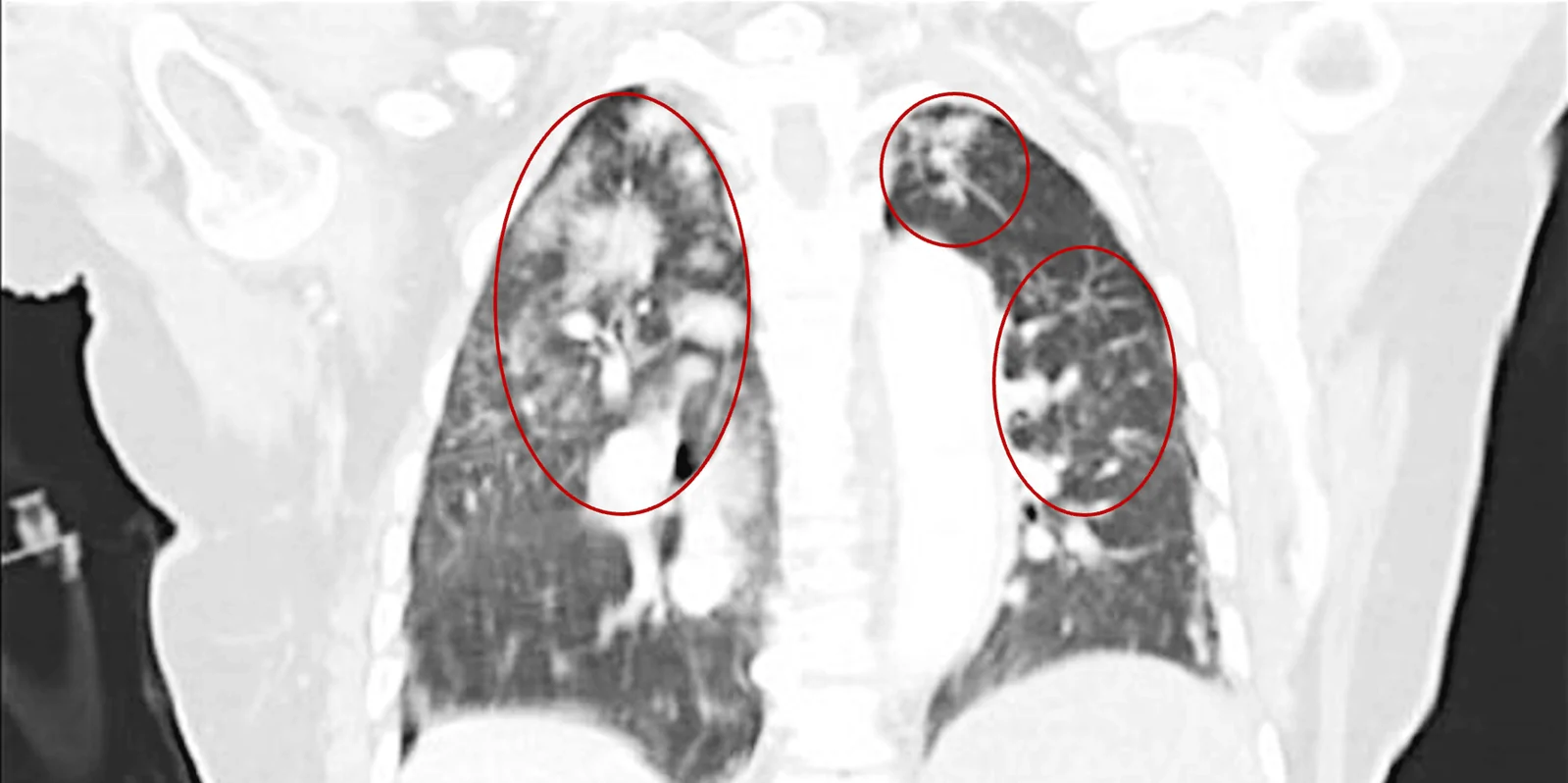
Initial chest computed tomography scan (coronal view) showing diffuse bilateral reticulonodular opacities and fibrotic changes (red circles).

**Figure 4:**
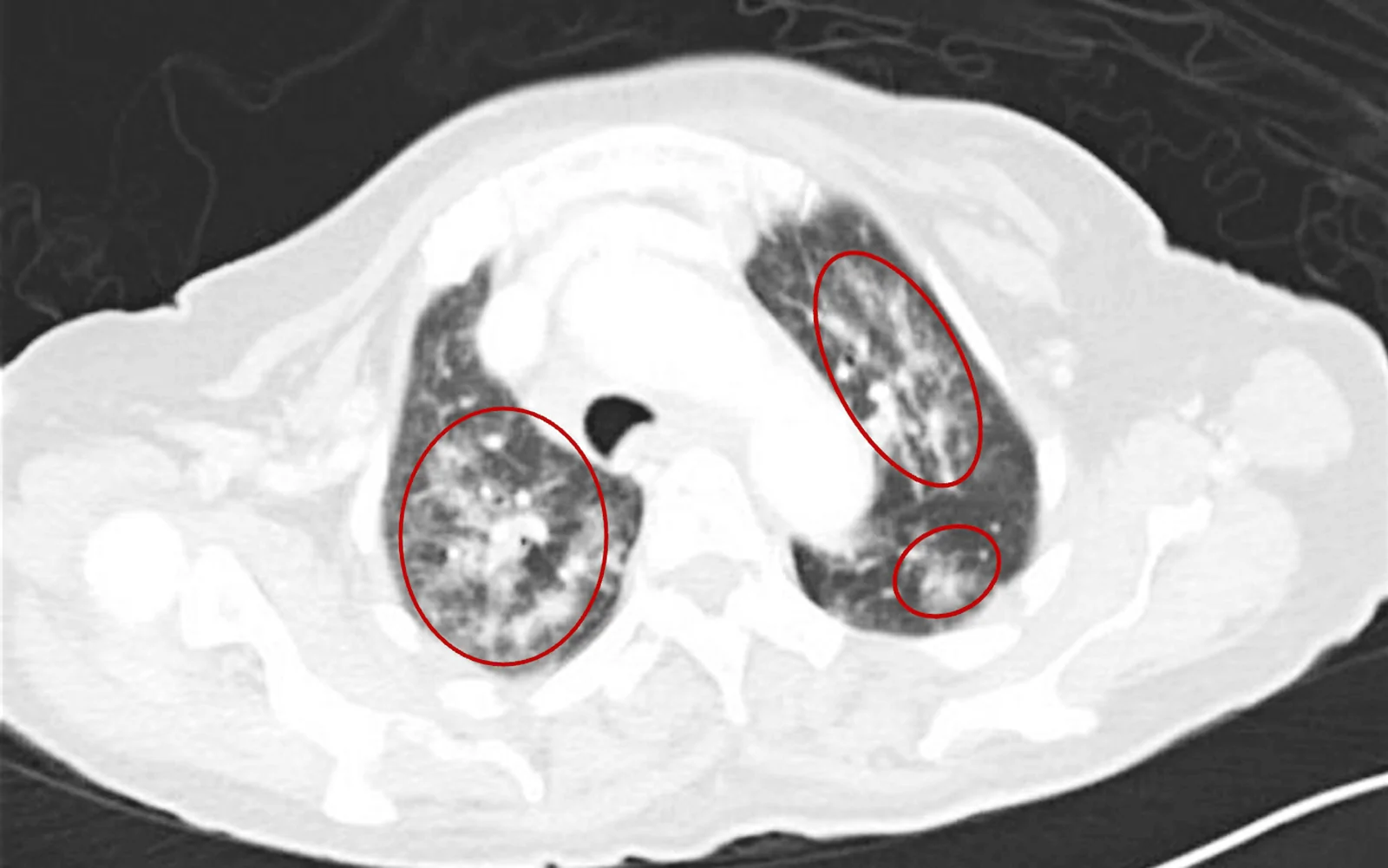
Initial chest computed tomography scan (axial view) showing diffuse bilateral reticulonodular opacities and fibrotic changes (red circles) in the upper lobes.

In view of the diffuse lymphadenopathy identified on radiological imaging, additional investigations were performed to further differentiate between lymphoproliferative malignancies and inflammatory or infectious causes. An autoimmune screening panel was requested as part of the diagnostic workup. 

An ultrasound-guided needle core biopsy was arranged, and multiple right inguinal lymph nodes were sampled. Subsequently, a bronchoscopy was performed to obtain a bronchoalveolar lavage (BAL) and transbronchial lung biopsy (TBLB) specimens. All samples were sent for cytological and histopathological analysis. The BAL cytology showed squamous cells, respiratory epithelial cells, and a few giant cells and lymphocytes, with no malignant cells observed. Moreover, the BAL culture was positive for *Candida albicans*. The Respiratory and Infectious Disease teams were consulted regarding the positive culture and associated chest radiographic findings, and oral fluconazole was initiated as per their recommendation.

Following a 21-day hospital stay, she was discharged on a course of oral fluconazole 400 mg daily for 14 days, with a two-week follow-up appointment in the clinic. The autoimmune antibodies panel and histopathological analysis of the biopsies were pending at the time of discharge and were scheduled for review in the outpatient setting.

Two-week follow-up

Two weeks after discharge, the patient was reviewed in the General Medicine outpatient clinic following the availability of the histopathological results. The histopathology report of the needle core biopsy of the right inguinal lymph nodes demonstrated extensive granulomatous inflammation with numerous non-caseating granulomas and frequent multinucleated giant cells. No cytological evidence of atypia or malignancy was observed. Moreover, Ziehl-Neelsen (ZN) staining was negative for acid-fast bacilli.

Similarly, the histopathological report of the transbronchial biopsy also demonstrated numerous non-caseating granulomas (Figures 5, 6) with occasional multinucleated giant cells within the subepithelium, accompanied by a mild chronic inflammatory cell infiltrate. There was no evidence of dysplasia or malignancy, and ZN staining was negative for acid-fast bacilli.

**Figure 5:**
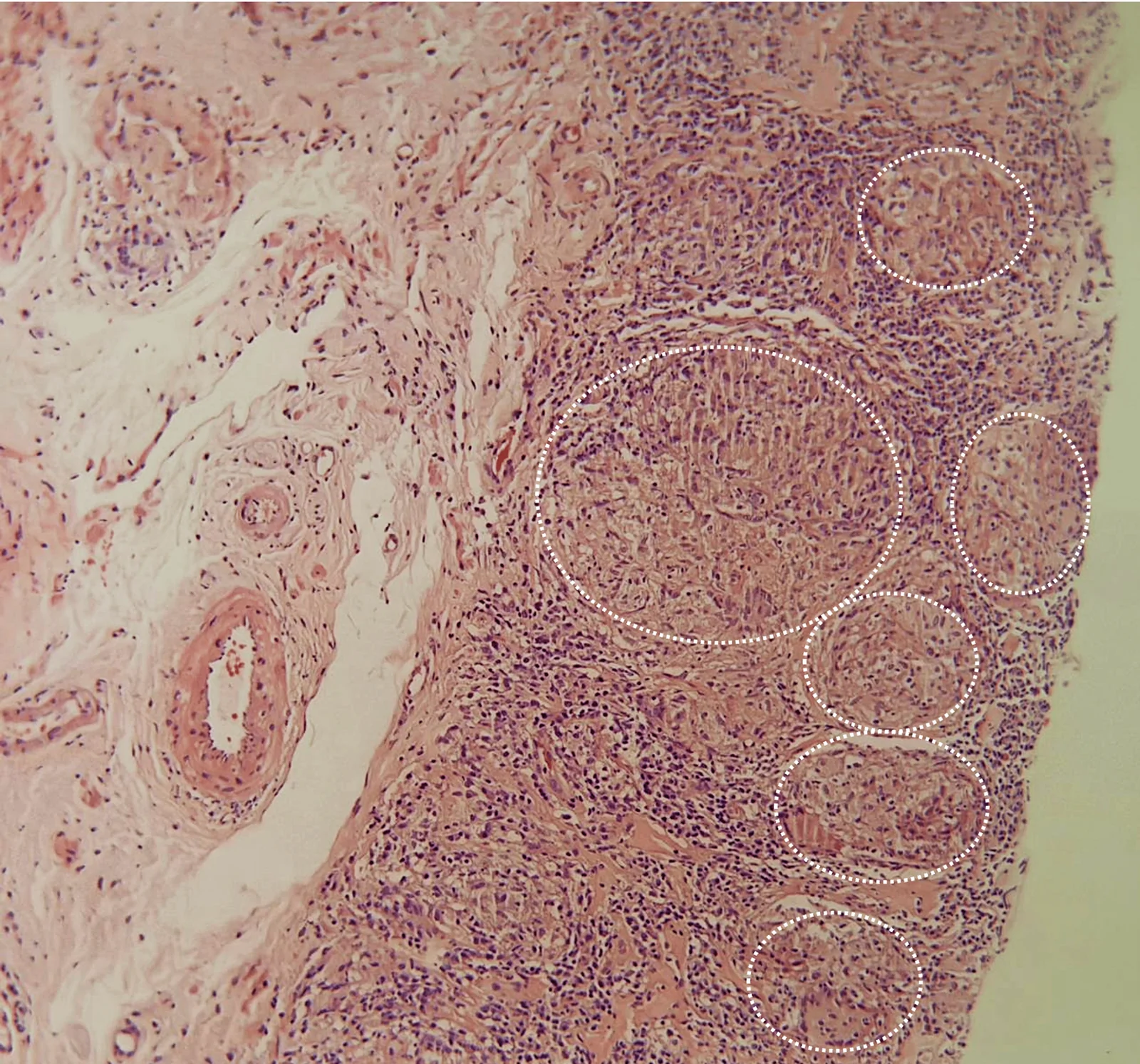
Lymph node histopathology slide demonstrating non-caseating granulomas (white dotted circles) on hematoxylin and eosin staining (×20 magnification).

**Figure 6:**
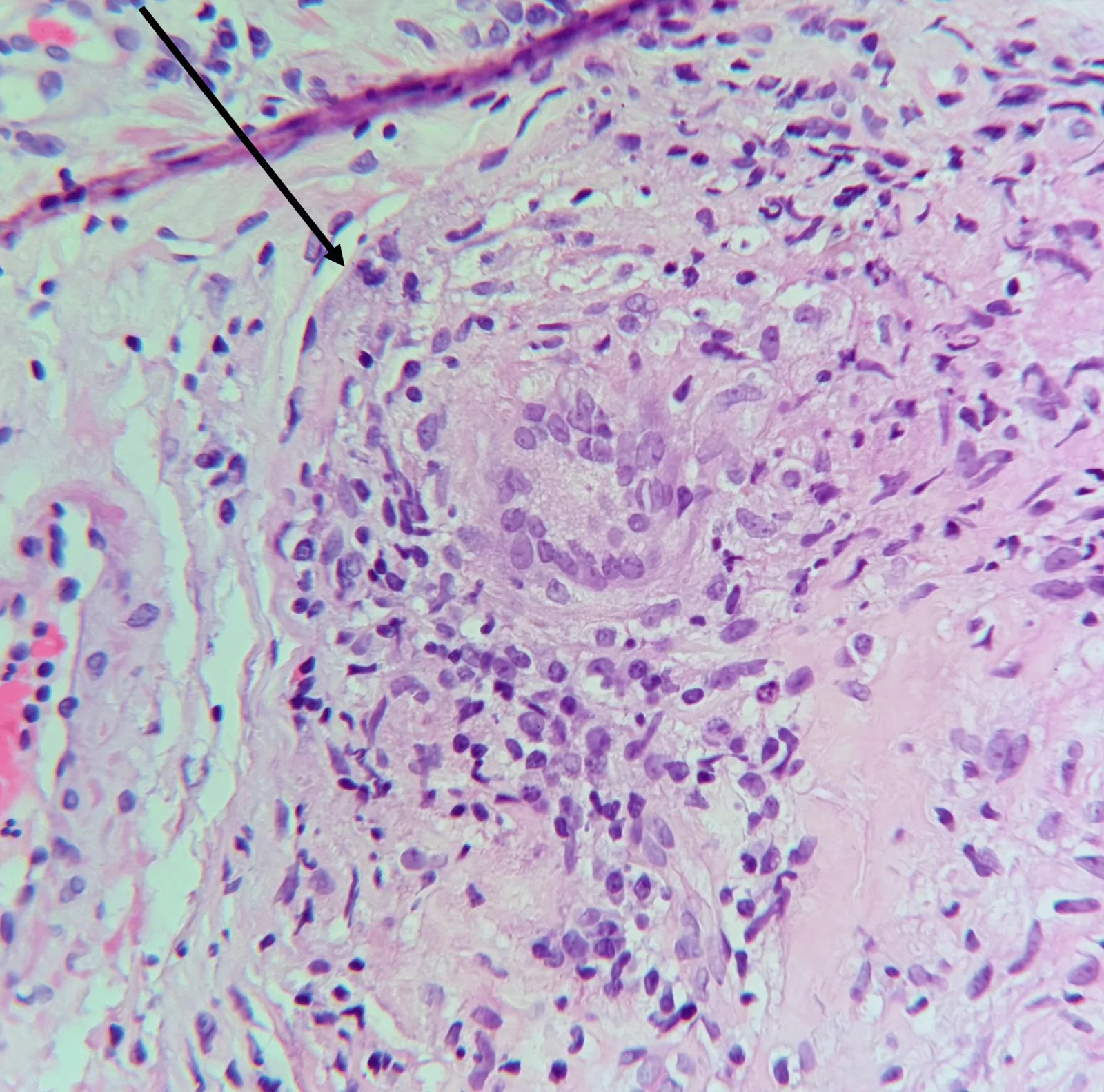
Lymph node histopathology slide showing non-caseating granuloma (black arrow) on hematoxylin and eosin staining (×40 magnification).

Given the histopathological findings of non-caseating granulomatous lymphadenitis, absence of malignant cells, and negative ZN staining, a diagnosis of sarcoidosis was established. A serum angiotensin-converting enzyme (ACE) level was requested before initiating corticosteroids to further corroborate the diagnosis. The findings were discussed with the patient and her family, and treatment was initiated with prednisolone 40 mg daily for four weeks. A gradual taper of 5 mg per week was planned thereafter, with dose adjustments guided by her clinical response and disease progression. Following multidisciplinary discussion, the Respiratory team concurred with the treatment plan and recommended a repeat high-resolution CT (HRCT) scan of the chest one month after initiation of corticosteroid therapy to assess treatment response.

Additionally, the results of the previously requested autoimmune screening panel were available for review and are summarised in Table 2. In view of the positive anti-Ro/SSA antibody, the patient was referred to the Rheumatology outpatient clinic to evaluate for possible coexisting Sjögren syndrome. Repeat autoimmune serological testing again demonstrated isolated anti-Ro/SSA antibody positivity. 

**Table 2:** Autoimmune antibody screening results at initial and repeat assessment

Autoimmune antibodies screening panel				
Parameter	Initial		Repeated	
	Titre value	Result	Titre value	Result
Anti-centromere IgG	0.5	Negative	0.5	Negative
Anti-histone IgG	13.5	Negative	7.3	Negative
Anti-Jo-1 IgG	1.8	Negative	0.6	Negative
Anti-ribosomal P IgG	0.7	Negative	0.5	Negative
Anti-Scl-70 IgG	0.6	Negative	0.5	Negative
Anti-La/SSB IgG	7.0	Negative	4.3	Negative
Anti-Ro/SSA IgG	43.2	Positive	20.5	Slightly positive
Anti-Smith IgG	1.3	Negative	0.5	Negative

The patient denied classical sicca symptoms, including xerostomia and xerophthalmia. In the absence of clinical features or additional serological findings supporting a diagnosis of Sjögren syndrome or another overlapping rheumatological disorder, no further Rheumatology follow-up was planned.

The patient was scheduled for a follow-up appointment in the General Medicine clinic one month later to review the HRCT findings and serum ACE level. She was instructed to continue prednisolone 40 mg daily until that visit, with plans for subsequent dose tapering based on her clinical response and the results of the pending investigations. Additionally, a baseline dual-energy X-ray absorptiometry (DXA) scan was requested to assess bone mineral density in view of the increased risk of osteoporosis associated with corticosteroid therapy.

Six-week follow-up

At the six-week follow-up appointment, the patient was clinically well and tolerating corticosteroid therapy without reported adverse effects. She denied respiratory symptoms, including cough and dyspnea. Physical examination revealed equal bilateral air entry on chest auscultation, with no adventitious breath sounds.

She was also reviewed for symptoms associated with hypercalcaemia, including constipation, abdominal pain, urinary symptoms, bone pain, and low mood, all of which she denied. Repeat laboratory investigations at six weeks showed improvement, including a normal serum calcium level of 2.3 mmol/L and the PTH level remained suppressed at 0.89 pmol/L. Serial measurements of relevant laboratory parameters from presentation through follow-up are summarised below in Table 3. Furthermore, the serum ACE level was also within the normal range at 30 U/L (reference range: 20-70 U/L).

**Table 3:** Trend of laboratory parameters from presentation through six-week follow-up

Parameter	Reference range (units)	On presentation	At discharge	At two-week follow-up	At six-week follow-up
White blood cell count	3.4-9.6 (×10⁹/L)	7.8	7.1	7.0	7.4
Hemoglobin	11.6-15.0 (g/dL)	11.7	11.6	11.4	11.6
Platelets	150-400 (×10⁹/L)	366	412	301	303
Urea	3.2-8.2 (mmol/L)	10.5	3.2	6.1	6.7
Creatinine	45-90 (μmol/L)	127	66	59	55
Total protein	57-82 (g/L)	89	90	100	88
Albumin	32-46 (g/L)	39	38	42	34
Globulin	15-30 (g/L)	50	52	58	54
Alkaline phosphatase	56-116 (U/L)	97	116	113	106
Gamma-glutamyl transferase	≤38 (U/L)	33	30	31	30
Alanine aminotransferase	10-49 (U/L)	11	10	10	12
Electrolytes	-	Within normal range	Within normal range	Within normal range	Within normal range
Calcium	2.20-2.55 (mmol/L)	3.72	2.25	2.44	2.30
Phosphorus	0.75-1.65 (mmol/L)	1.2	0.77	1.1	0.76
Vitamin D	≥50 (nmol/L)	84	-	-	80
Parathyroid hormone	1.96-9.33 (pmol/L)	0.76	-	-	0.89
Erythrocyte sedimentation rate	≤20 (mm/hour)	120	115	120	40
C-reactive protein	≤9 (mg/L)	4	3	4	4

The repeat HRCT scan of the chest demonstrated significant interval regression of the previously observed diffuse bilateral reticulonodular opacities (Figures 7, 8). Bilateral upper lobe fibrotic bands were noted, with no evidence of pleural effusion. Multiple prominent but stable mediastinal lymph nodes were identified, measuring up to 1.3 cm in short-axis diameter at the left hilar region and 1.0 cm in the upper paratracheal region. Mild dilatation of the pulmonary trunk and main pulmonary arteries was also noted. Overall, the repeat HRCT demonstrated significant radiological improvement following corticosteroid therapy.

**Figure 7:**
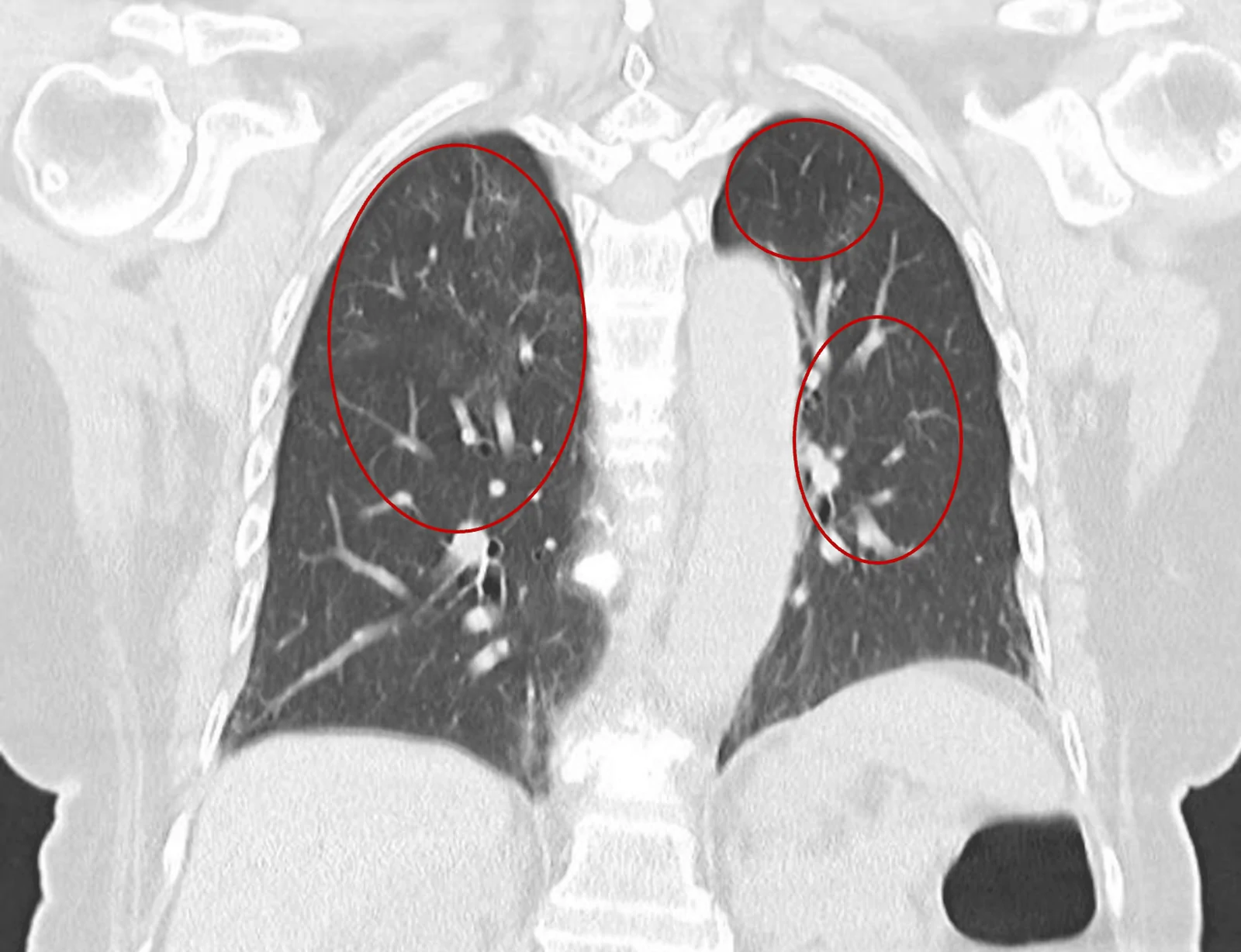
Six-week follow-up high-resolution computed tomography scan of the chest (coronal view) showing significant regression of previously observed diffuse bilateral reticulonodular opacities (red circles).

**Figure 8:**
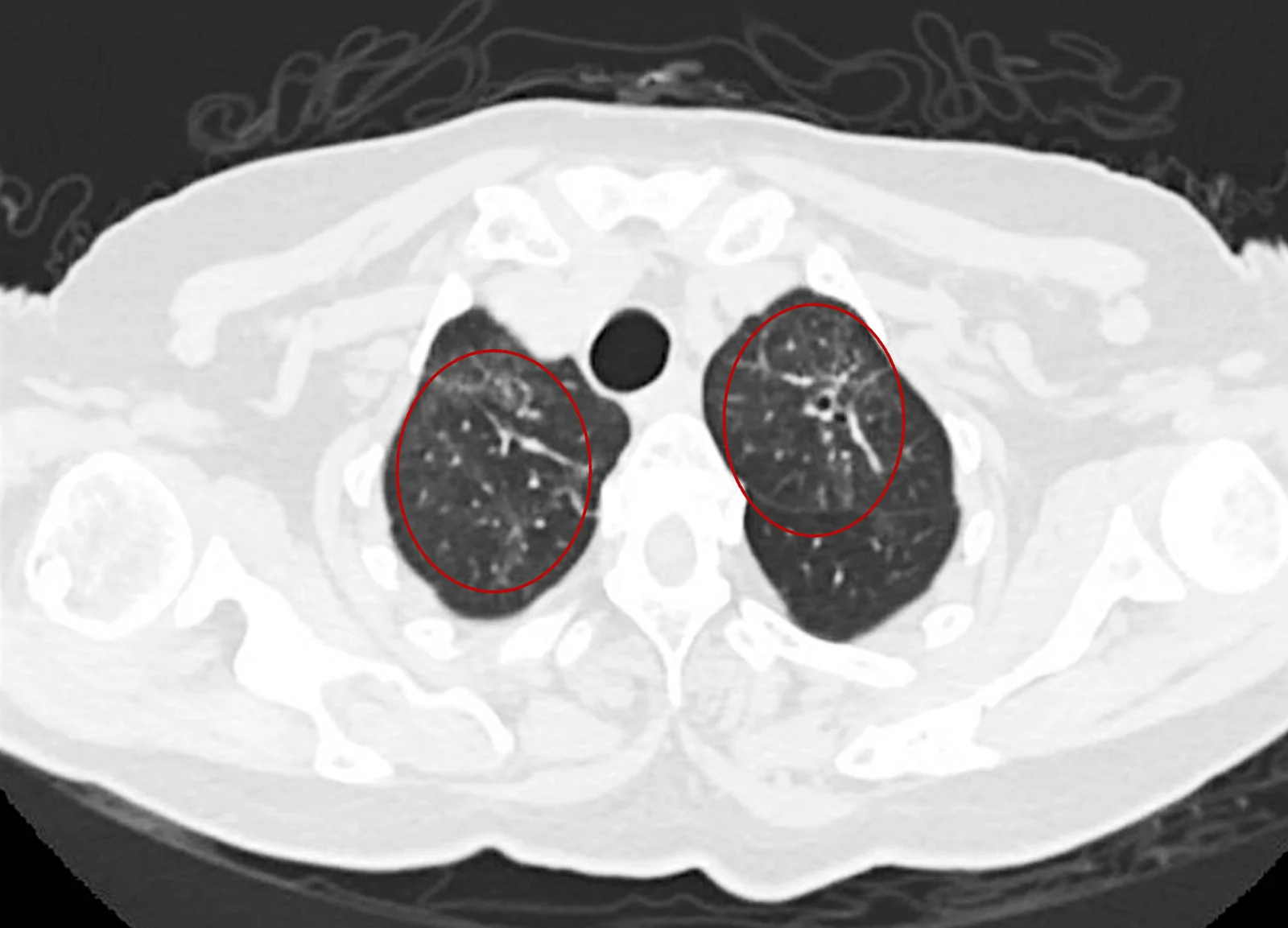
Six-week follow-up high-resolution computed tomography scan of the chest (axial view) showing significant regression of diffuse bilateral reticulonodular opacities (red circles) previously observed in the upper lobes.

Following a favourable response to one month of oral prednisolone 40 mg daily, including symptomatic improvement, stable biochemical parameters, and radiological regression, a gradual tapering regimen was initiated. The prednisolone dose was reduced by 5 mg weekly until a dose of 10 mg daily was reached. After weighing the risks and benefits of continued corticosteroid therapy, a maintenance dose of prednisolone 10 mg daily was recommended, with follow-up in the General Medicine and Respiratory clinics after three months to reassess her clinical status and consider further dose reduction or discontinuation.

## Discussion

Sarcoidosis is a multisystem granulomatous disorder characterised by marked variability in organ involvement and clinical presentation, contributing to its diagnostic complexity. To our knowledge, previous literature reviews have described cases of sarcoidosis in patients up to 81 years of age [6], while a more recent cohort study of elderly-onset sarcoidosis (EOS) included patients up to 84 years of age [7]. The diagnosis of histopathologically confirmed sarcoidosis in our 83-year-old patient contributes to the limited literature on elderly-onset disease. Despite its rarity in advanced age, sarcoidosis should remain in the differential diagnosis of elderly patients presenting with constitutional symptoms, hypercalcaemia, and lymphadenopathy, particularly when these features raise concern for malignancy.

Differences in clinical presentation between younger-onset sarcoidosis (YOS) and EOS have been increasingly recognised in the literature. Sarcoidosis is frequently identified incidentally in younger individuals, many of whom are asymptomatic at diagnosis [8]. Conversely, EOS is seldom asymptomatic and more often associated with overt clinical manifestations [8]. While the classic respiratory manifestations of sarcoidosis, including dyspnea and chronic dry cough, occur in both YOS and EOS, extrapulmonary involvement appears to be more prominent among patients older than 65 years [8]. Elderly patients also appear more likely to exhibit constitutional symptoms, including fatigue, anorexia, and weight loss, with reported prevalence rates ranging from 33% to 43%, compared with approximately 6% to 16% in younger individuals [8,9]. These non-specific symptoms may obscure the diagnosis in elderly patients by mimicking malignancy and other age-related disorders.

Evidence supporting these differences was demonstrated in a 2018 single-centre retrospective study from Turkey that evaluated the demographic, laboratory, radiological, and clinical characteristics of 131 patients with sarcoidosis [5]. Compared with patients with YOS, those with EOS more frequently presented with constitutional symptoms, particularly fatigue (75%), and exhibited a higher prevalence of extrapulmonary manifestations, including ocular (20% vs. 13.5%) and cutaneous involvement (50% vs. 44.1%) [5]. The EOS cohort also had a greater burden of comorbidities [5]. Notably, diagnostic delay was shorter among elderly patients, which may reflect their increased propensity to present with constitutional symptoms that prompt more extensive diagnostic investigations [5]. Despite these clinical differences, radiological findings and laboratory parameters, including inflammatory markers, were comparable between the two groups [5].

Hypercalcaemia is a well-recognised metabolic complication of sarcoidosis, occurring in approximately 5%-10% of patients and occasionally representing the initial manifestation of the disease [10]. The underlying mechanism involves excessive extrarenal synthesis of calcitriol (1,25-dihydroxyvitamin D) by activated macrophages within granulomatous lesions, leading to increased intestinal calcium absorption and contributing to disturbances in calcium homeostasis [10]. However, the absence of a serum calcitriol measurement represents a limitation in establishing the underlying mechanism of hypercalcaemia in our case. Although the suppressed PTH level and normal vitamin D (25-hydroxyvitamin D) level supported a PTH-independent aetiology, extrarenal calcitriol production could not be definitively assessed.

In elderly patients, the coexistence of severe symptomatic hypercalcaemia, lymphadenopathy, and constitutional symptoms, such as anorexia, weight loss, fatigue, and fever, should raise suspicion for an underlying malignancy. The differential diagnosis includes hematologic and lymphoproliferative malignancies, such as multiple myeloma and lymphoma, as well as primary hyperparathyroidism and solid organ malignancies, including lung, breast, prostate, and renal cell carcinomas [11]. In malignancy-associated hypercalcaemia, serum PTH levels are typically suppressed or undetectable, and hypercalcaemia is often a late manifestation of advanced disease and is associated with an adverse prognosis [11]. Therefore, distinguishing malignancy-associated hypercalcaemia from sarcoidosis-related hypercalcaemia is particularly important in older adults, given the overlapping clinical features of these conditions.

The diagnosis of sarcoidosis is established through the integration of clinical, radiological, and histopathological findings after exclusion of alternative diagnoses, including infectious, malignant, and other granulomatous disorders [12]. In the present case, the diagnosis was supported by the presence of prominent lymphadenopathy with diffuse pulmonary involvement on imaging, together with non-caseating granulomas identified in both inguinal lymph nodes and transbronchial biopsy specimens. Negative ZN staining and the absence of cytological evidence of malignancy further supported the diagnosis.

Although serum ACE levels are frequently elevated in patients with sarcoidosis, their diagnostic utility is limited by low sensitivity, with elevated levels reported in approximately 60% of cases [13]. In our patient, the serum ACE level remained within the normal range despite histopathological confirmation of sarcoidosis, further illustrating the limitations of this biomarker. Therefore, serum ACE levels should be regarded as an adjunctive rather than definitive diagnostic tool and interpreted within the context of the clinical presentation, imaging findings, and histopathological evidence. 

Systemic corticosteroids remain the cornerstone of treatment for symptomatic sarcoidosis, particularly in patients with significant organ involvement or clinically relevant complications such as hypercalcaemia [10,14]. Corticosteroid therapy reduces granulomatous inflammation and suppresses macrophage-mediated extrarenal calcitriol production, thereby improving calcium homeostasis [10,14]. Similar to our patient, treatment with oral prednisolone results in resolution of hypercalcaemia, improvement in constitutional symptoms, and significant radiological regression of pulmonary abnormalities, demonstrating a favourable therapeutic response.

## Conclusions

This case illustrates the importance of resisting diagnostic anchoring in elderly patients presenting with hypercalcaemia and constitutional symptoms. While malignancy appropriately remains a leading consideration, alternative inflammatory and granulomatous disorders may present with overlapping clinical features. When a common diagnosis appears to explain the presenting features, maintaining a broad differential and systematically evaluating alternative etiologies are essential to avoid premature diagnostic closure. The diagnosis of sarcoidosis in this patient required integration of biochemical, radiological, and histopathological findings, supported by a comprehensive multidisciplinary approach. Recognition of such atypical presentations may increase awareness of elderly-onset sarcoidosis and facilitate timely diagnosis and appropriate management.
